# Association of Newborn Telomere Length With Blood Pressure in Childhood

**DOI:** 10.1001/jamanetworkopen.2022.25521

**Published:** 2022-08-05

**Authors:** Dries S. Martens, Hanne Sleurs, Yinthe Dockx, Leen Rasking, Michelle Plusquin, Tim S. Nawrot

**Affiliations:** 1 Centre for Environmental Sciences, Hasselt University, Hasselt, Belgium; 2 Research Unit Environment and Health, Department of Public Health and Primary Care, University of Leuven, Leuven, Belgium

## Abstract

**Importance:**

Adult telomere length (TL) is a biological marker of aging associated with vascular health. TL at birth is associated with later life TL and may contain early biological information of later life cardiovascular health and disease.

**Objective:**

To evaluate whether newborn TL is associated with early life blood pressure differences in childhood.

**Design, Setting, and Participants:**

This cohort study was part of the ENVIRONAGE (Environmental Influence on Aging in Early Life) study, a birth cohort of Belgian mother-child pairs with recruitment at birth and a median follow-up of 4.5 years conducted between October 2014 and July 2021. Participants included for analysis provided full data for evaluation at follow-up visit. Data analysis was conducted between August and September 2021.

**Main Outcomes and Measures:**

Cord blood and placental average relative TL were measured at birth using quantitative polymerase chain reaction (qPCR). Systolic, diastolic, and mean arterial pressure (MAP) were evaluated at follow-up. High childhood blood pressure (standardized for child age, sex, and height) was defined following the 2017 American Academy of Pediatrics guidelines. Multivariable adjusted linear and logistic regression models were used to associate newborn TL and blood pressure indicators in childhood.

**Results:**

This study included 485 newborn children (52.8% girls) with a mean (SD) age of 4.6 (0.4) years at the follow-up visit. Newborn TL was associated with lower blood pressure in childhood. A 1-IQR increase in cord blood TL was associated with a –1.54 mm Hg (95% CI, –2.36 to –0.72 mm Hg) lower diastolic blood pressure and –1.18 mm Hg (95% CI, –1.89 to –0.46 mm Hg) lower MAP. No association was observed with systolic blood pressure. Furthermore, a 1-IQR increase in cord blood TL was associated with lower odds of having high blood pressure at the age of 4 to 6 years (adjusted OR, 0.72; 95% CI, 0.53 to 0.98). In placenta, a 1-IQR increase in TL was associated with a –0.96 mm Hg (95% CI, –1.72 to –0.21 mm Hg) lower diastolic, –0.88 mm Hg (95% CI, –1.54 to –0.22 mm Hg) lower MAP, and a lower adjusted OR of 0.69 (95% CI, 0.52 to 0.92) for having a high blood pressure in childhood.

**Conclusions and Relevance:**

In this prospective birth cohort study, variation in early life blood pressure at school-age was associated with TL at birth. Cardiovascular health may to some extent be programmed at birth, and these results suggest that TL entails a biological mechanism in this programming.

## Key Points

**Question:**

Is newborn telomere length (TL) associated with blood pressure in childhood?

**Findings:**

In this birth cohort study including 485 newborns, participants with a longer birth TL had a significantly lower diastolic and mean arterial pressure at the age of 4 to 6 years. Furthermore, longer newborn TL was significantly associated with lower odds of having high childhood blood pressure.

**Meaning:**

These results suggest that TL at birth associates with early life vascular health and that newborn TL may be a programmed molecular mediator containing genetic and prenatal environmental exposure information contributing to vascular health later in life.

## Teaser

This cohort study of Belgian mother-child pairs examines the association of newborn telomere length with blood pressure in childhood.

## Introduction

Cardiovascular diseases in adults often find their roots in risk factors operative early in life. Blood pressure substantially rises with aging across the life span. Blood pressure tracks from childhood to adolescence and adulthood, and high blood pressure is associated with cardiovascular disease in later life.^1,2^

Telomeres are nucleoprotein complexes capping the end of our chromosomes that shorten due to cell division and advancing age.^3^ Telomere length (TL) is considered a biological indicator of age that relates to cardiovascular disease (CVD) and mortality in adult humans, independent of chronological age.^4^ The telomere biological system is an important molecular mechanism that may be causally related to cardiovascular aging,^5^ an effect supported by experimental^6^ and human population-based studies.^7^ Dysfunctional telomere-related senescence of the vascular wall and impaired endothelial functioning may explain why a shortened TL is observed in hypertensive individuals.^8,9^

At birth, TL is highly variable and strongly projects towards later life TL.^10^ Furthermore, TL tracks over different age segments in life,^11,12^ showing that TL-related health conditions observed later in life may find their origin at birth to some extent. This highlights telomeres as an important molecular explanation in the fetal programming of health and disease hypothesis.^13^ Recent advances have been made to understand this wide variation of TL at birth, with a main focus on prenatal environmental and lifestyle factors,^14,15,16,17^ although the consequences of this early variation in relation to later life (cardiovascular) health outcomes are underexplored. The first evidence of newborn TL and later life cardiovascular health showed that after a 40-year follow-up in 144 individuals a short birth TL is associated with an increased risk of projected atherosclerotic lesions by the Pathobiological Determinants of Atherosclerosis in Youth (PDAY) score.^18^ Further preliminary evidence by Kaali and colleagues^19^ suggests that cord blood TL may relate to systolic blood pressure (SBP) in children at the age of 4 years (in a cohort with 97 children). To our knowledge, no large population-based studies have been evaluating the correlation of newborn TL with childhood blood pressure measures as a major adult risk factor for CVD. Therefore, we studied whether newborn TL is associated with blood pressure measures in childhood as phenotypical indicators of early cardiovascular aging. Cord blood and placental TL at birth were studied in the ENVIRONAGE birth cohort in relation to blood pressure indicators (SBP, diastolic blood pressure [DBP], and mean arterial pressure [MAP]) in early childhood (ie, children ages 4 to 6 years).

## Methods

### Study Population

**Figure 1.**
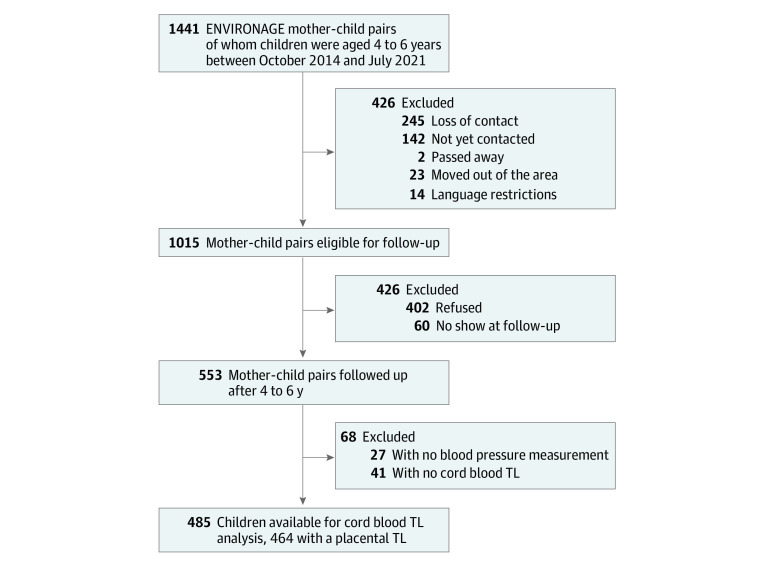
Flowchart Describing Included ENVIRONAGE Participants

The ongoing ENVIRONAGE study was initiated in 2010 in Limburg, Belgium. Mother-newborn pairs are recruited at birth and are invited for a follow-up around the age of 4 years (Figure 1). The study complies with the Helsinki Declaration and has been approved by the ethics committees of Hasselt University and East-Limburg Hospital. Mothers provided written informed consent at the 2 study phases. This study was conducted following the Strengthening the Reporting of Observational Studies in Epidemiology (STROBE) guidelines.

At birth the selection criteria was the mother’s ability to fill out questionnaires in Dutch. Between October 2014 and July 2021 in total 1441 participants were aged between 4 and 6 years, of whom 1015 met the criteria for follow-up (including could be contacted, not moved out of the area, not passed away, could communicate in Dutch). A total of 553 child-mother pairs (54.5%) were followed-up, of whom 485 children had a blood pressure measurement and TL at birth and formed the basis population of the current analysis. The studied ENVIRONAGE population is demographically comparable with the not-analyzed fraction of the cohort and representative of the reproductive segment of the population at large^20^ for maternal age, parity, sex, gestational age, and birth weight, but did slightly differ in maternal education and newborn ethnicity (eTable 1 in the Supplement).

### Measures

At birth, information on gestational age (in weeks, estimated based on the last menstrual cycle and first fetal ultrasound examination), birth weight (in grams), maternal prepregnancy body mass index (BMI; calculated as weight in kilograms divided by height in meters squared), and history of gestational hypertension and preeclampsia are collected from medical records. Via questionnaires filled out by the mothers in the maternity ward, information on maternal age, education (categorized by International Standard Classification of Education^21^ coding: low when no diploma was obtained, middle when a secondary school diploma was obtained, and high when a high school or university diploma was obtained), smoking (nonsmokers, stopped before pregnancy, and continued smokers), and newborn ethnicity (European, non-European) was collected.

At follow-up visits conducted between 4 and 6 years later, body anthropometrics of the children including height (to the nearest centimeter) and weight (to the nearest 0.1 kg) were measured. BMI and childhood overweight (including obesity) were defined using age- and sex-specific BMI cut-offs according to the International Obesity Task Force.^22^ Detailed questionnaires were filled out by the mothers to obtain household and lifestyle information. For this study, data on household smoking status (ie, child exposure to second-hand smoke) during the follow-up period was collected (absent when none of the parents smoked in the home, and present when at least 1 parent smoked in the home). Based on the date of follow-up, a seasonal scale was calculated (winter, December 21 to March 20; spring, March 21 to June 20; summer, June 21 to September 20; autumn, September 21 to December 20).

### Follow-up Blood Pressure Assessment

Child and maternal blood pressure were measured with an automated upper-arm blood pressure monitor (Omron Corporation) equipped with a cuff adapted to the arm size of children. Measurements were performed by a standardized method, as described by the European Society of Hypertension.^23^ After 5 minutes of rest in the supine position, a trained observer obtained 5 consecutive readings of the SBP and DBP at 1-minute intervals. Mean SBP and DBP were based on the mean of the last 3 readings. MAP was calculated as DBP plus one-third of pulse pressure (the difference between SBP and DBP).

### Newborn TL Measurement

Placental and cord blood TL were measured as described previously.^24^ Information on sample collection, processing, DNA extraction, DNA quantification and integrity evaluation, quantitative polymerase chain reaction (qPCR) details, and quality controls are provided in eMethods 1 in the Supplement. The reliability of our qPCR method was assessed using an intraclass correlation coefficient (ICC).^25^ The inter-assay ICC was 0.94 (95% CI, 0.81-0.97) and the intra-assay ICC was 0.95 (95% CI, 0.95-0.96).

### Statistical Analysis

All data management and statistical analyses were done using SAS version 9.4 (maintenance level 5; SAS Institute Inc). All performed statistical tests were 2-sided and a *P* value <.05 was considered statistically significant. Participants’ characteristics were presented as means (with SDs) or frequency. Pearson correlations and single and multivariable-adjusted linear and logistic regression models were applied.

In a first step, the association between cord blood and placenta TL (independent variables) and SBP, DBP, and MAP (dependent variables) in children ages 4 to 6 years were analyzed using separate linear regression models (continuous analysis). First, we adjusted our models for a priori selected prenatal and perinatal covariates that may be associated with both the dependent and independent variables, including newborn sex, ethnicity, gestational age, birth weight, maternal age, maternal prepregnancy BMI, maternal education, maternal smoking during pregnancy, and maternal gestational hypertension and preeclampsia. Second, we further adjusted for postnatal factors between ages 4 and 6 years, including child age, child weight, child height, and household smoke exposure during childhood. To evaluate the robustness of our models we stratified our models for sex, excluded preterm births (gestational age below 37 weeks) and children who were overweight (according to the International Obesity Task Force cut-offs). Furthermore, we adjusted the models for child BMI instead of child weight and height, adjusted for date and season of the follow-up, and additionally adjusted the model for the corresponding maternal blood pressure indicator. Estimates are presented as the difference in blood pressure for a 1-IQR increase in cord blood or placental TL.

In a second step, we evaluated whether newborn TL was associated with the odds of having a high blood pressure in childhood. Because no reference values are currently available for high blood pressure in European children, current guidelines^26^ recommend using US reference values. Therefore, we used the 2017 American Academy of Pediatrics (AAP) guidelines for defining high blood pressure.^27^ Age-, sex-, and height-specific BP percentiles were calculated for SBP and DBP and referred to as high when above the 90th percentile (including elevated, stage 1 hypertension and stage 2 hypertension BP levels as defined by the 2017 AAP) (eTable 2 in the Supplement). Logistic regression models were applied adjusting for the aforementioned covariates. Odds ratios with 95% CIs are reported for a 1-IQR increase in cord blood or placental TL.

## Results

### Study Population

Of the 485 ENVIRONAGE participants included in analysis, newborns had a mean (SD) gestational age of 39.1 (1.6) weeks and a birth weight of 3393 (490) grams; 256 (52.8%) were girls, and 26 (5.4%) were of non-European ethnicity (Table 1). Mothers had a mean (SD) age of 30.2 (4.1) years at delivery and a prepregnancy BMI of 24.3 (4.4). Most mothers were highly educated (319 [65.8%]) and nonsmokers (329 [67.8%]). The median follow-up was 4.5 years (5th to 95th percentile, 4.1-5.5 years), and children weighed a mean 18.8 kg and were 107.9 cm tall. Mean (SD) SBP, DBP, and MAP were 98.3 (7.9), 55.4 (6.9), and 69.7 (6.0) mm Hg for children on follow-up, respectively. Mothers had an SBP, DBP, and MAP of 116.7 (11.6), 69.7 (8.8), and 85.4 (9.2) mm Hg. Cord blood (range, 0.54-1.70 mm Hg) and placenta TL (range, 0.38-2.01 mm Hg) had an IQR of 0.26 and 0.32, respectively. Cord blood and placental TL correlated (Pearson *r* = 0.40; *P* < .001).

**Table 1.** Characteristics of 485 ENVIRONAGE Participants

Characteristic	Participants, No. (%)
**Newborn**	
Sex	
Girls	256 (52.8)
Boys	229 (47.2)
Ethnicity	
European	459 (94.6)
Non-European	26 (5.4)
Gestational age, mean (SD), wks	39.1 (1.6)
Birth weight, mean (SD), g	3393 (490)
**Child at follow-up**	
Age, mean (SD), y	4.6 (0.4)
Weight, mean (SD), kg	18.8 (2.6)
Height, mean (SD), cm	107.9 (5.1)
BMI, mean (SD)	16.1 (1.3)
Overweight^a^	66 (13.6)
Obese^a^	9 (1.9)
Pressure, mean (SD), mm Hg	
Systolic	98.3 (7.9)
Diastolic	55.4 (6.9)
Mean arterial	69.7 (6.0)
AAP 2017 BP category^b^	
Normal	379 (78.2)
Elevated	49 (10.1)
Stage 1 hypertension	55 (11.3)
Stage 2 hypertension	2 (0.4)
Household smoke	
No	352 (72.6)
Yes	133 (27.4)
Season	
Winter	120 (24.7)
Spring	140 (28.9)
Summer	116 (23.9)
Autumn	109 (22.5)
**Mother**	
Age at delivery, mean (SD), y	30.2 (4.1)
Prepregnancy BMI, mean (SD)	24.3 (4.4)
Education	
Low	28 (5.8)
Middle	138 (28.4)
High	319 (65.8)
Smoking during pregnancy	
Nonsmoker	329 (67.8)
Stopped	106 (21.9)
Continued	50 (10.3)
Pregnancy hypertension plus preeclampsia	
No	461 (95.1)
Yes	24 (4.9)
Pressure at follow-up, mean (SD), mm Hg^c^	
Systolic	116.7 (11.6)
Diastolic	69.7 (8.8)
Mean arterial	85.4 (9.2)

Abbreviations: AAP, American Academy of Pediatrics; BMI, body mass index (calculated as weight in kilograms divided by height in meters squared); BP, blood pressure.

SI conversion factor: To convert BP to kilopascals, multiply by 0.133.

^a^ As defined by age- and sex-specific BMI cut-offs according to the IOTF.

^b^ As defined by 2017 AAP^27^ using age-, sex-, and height-specific BP percentiles (eTable 2 in the Supplement).

^c^ Maternal blood pressures available for 459 participants.

### Newborn TL and Child Blood Pressure Indicators

**Figure 2.**
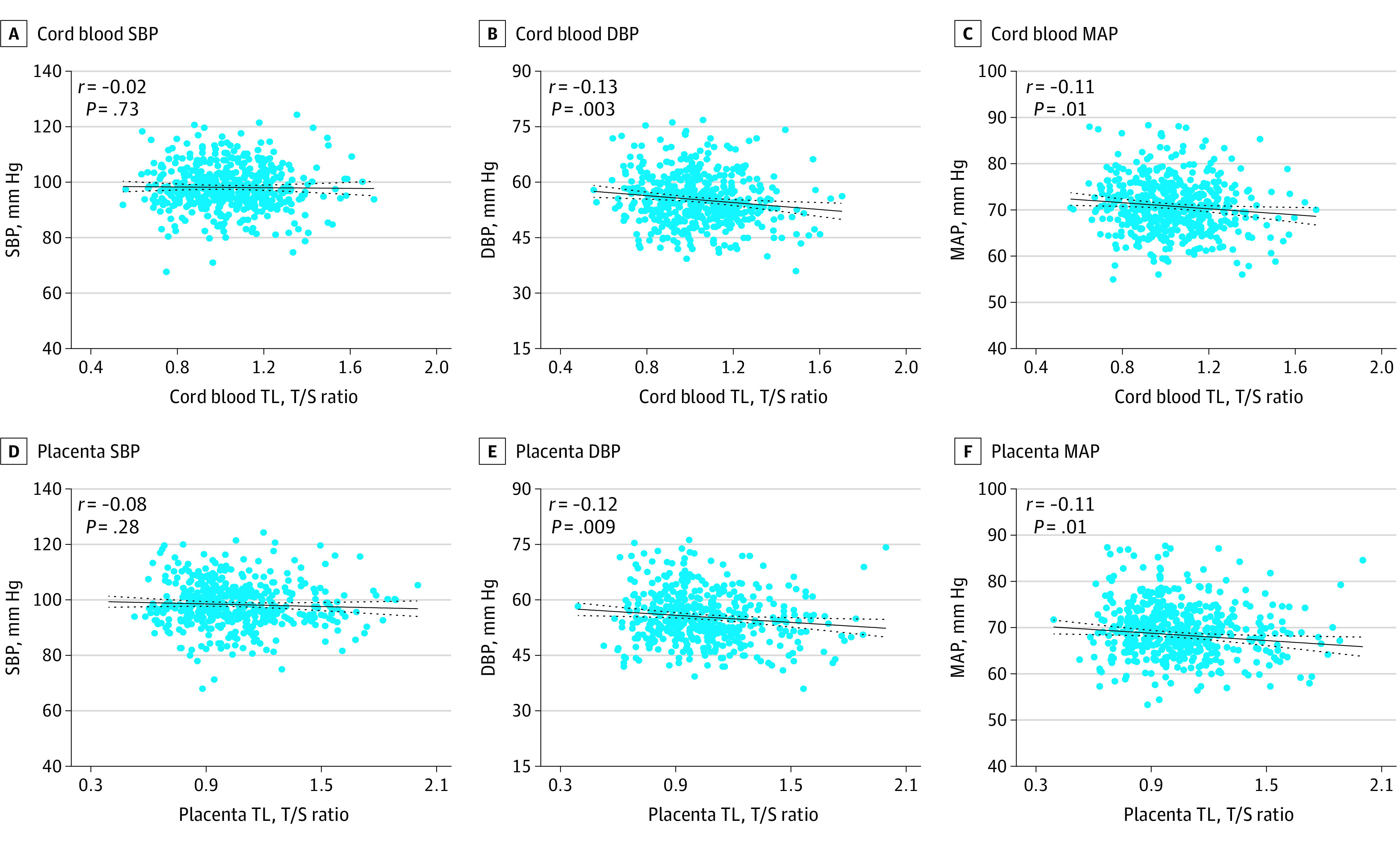
Pearson Correlation Between Newborn Telomere Length (TL) and Blood Pressure at Ages 4 to 6 Years Black lines indicate the unadjusted regression line with 95% CIs (dotted lines); DBP, diastolic blood pressure; MAP, mean arterial pressure; SBP, systolic blood pressure; T/S, telomere to single copy gene ratio. Results for cord blood (panels A, B, and C) include 485 children; for placenta (panels D, E, and F), 464 children.

Cord blood and placenta TL were negatively correlated with DBP (*r* = –0.13; *P* = .003 and *r* = –0.12; *P* = .009) and MAP (*r* = –0.11; *P* = .01 and *r* = –0.11; *P* = .01), but not with SBP (Figure 2). These associations were confirmed after adjustment for newborn sex, ethnicity, gestational age, birth weight, maternal age, maternal prepregnancy BMI, maternal education, maternal gestational smoking and maternal gestational hypertension/preeclampsia, child age, child weight, and child height at follow-up and household smoke exposure (Table 2). In these full-adjusted models, a 1-IQR increase in cord blood TL was associated with a –1.54 mm Hg (95% CI, –2.36 to –0.72 mm Hg; *P* < .001) and –1.18 mm Hg (95% CI, –1.89 to –0.46 mm Hg; *P* = .001) lower DBP and MAP, respectively, at the age of 4 to 6 years. Longer cord blood TL was not associated with SBP (per 1-IQR increase: –0.45 mm Hg; 95% CI, –1.40 to 0.50 mm Hg; *P* = .35). A 1-IQR increase in placental TL was associated with a –0.96 mm Hg (95% CI, –1.72 to –0.21 mm Hg; *P* = .01) and –0.88 mm Hg (95% CI, –1.54 to –0.22 mm Hg; *P* = .009) lower DBP and MAP, respectively. Longer placental TL was not associated with lower SBP (per 1-IQR increase: –0.71 mm Hg; 95% CI, –1.59 to 0.16 mm Hg; *P* = .10). Comparable regression estimates were observed when stratifying for boys and girls, or when excluding preterm births or children that were overweight (eTable 3 in the Supplement). Further adjustment for date and season of follow-up slightly decreased the regression estimates, and additional adjustment for childhood BMI or maternal blood pressure at follow-up did not alter the aforementioned results (eTable 3 in the Supplement).

**Table 2.** Association Between Newborn Telomere Length and Blood Pressure in Children Aged 4 to 6 Years

Blood pressure indices	Cord blood TL (n = 485)		Placenta TL (n = 464)	
	Difference (95% CI), mm Hg^a^	*P* value	Difference (95% CI), mm Hg^a^	*P* value
Systolic blood pressure				
Unadjusted model	–0.17 (–1.12 to 0.79)	.73	–0.49 (–1.38 to 0.40)	.28
Model 1^b^	–0.28 (–1.24 to 0.69)	.58	–0.53 (–1.43 to 0.37)	.25
Model 2^c^	–0.45 (–1.40 to 0.50)	.35	–0.71 (–1.59 to 0.16)	.10
Diastolic blood pressure				
Unadjusted model	–1.22 (–2.04 to –0.40)	.003	–1.01 (–1.77 to –0.25)	.009
Model 1^b^	–1.46 (–2.28 to –0.64)	<.001	–0.93 (–1.68 to –0.17)	.02
Model 2^c^	–1.54 (–2.36 to –0.72)	<.001	–0.96 (–1.72 to –0.21)	.01
Mean arterial pressure				
Unadjusted model	–0.87 (–1.59 to –0.16)	.02	–0.83 (–1.50 to –0.17)	.01
Model 1^b^	–1.07 (–1.78 to –0.35)	.004	–0.80 (–1.46 to –0.13)	.02
Model 2^c^	–1.18 (–1.89 to –0.46)	.001	–0.88 (–1.54 to –0.22)	.009

Abbreviations: BMI, body mass index (calculated as weight in kilograms divided by height in meters squared); TL, telomere length.

^a^ Estimates presented as a difference in blood pressure indicator in mm Hg for a 1-IQR increase cord blood or placental TL.

^b^ Adjusted for newborn sex, ethnicity, gestational age, birth weight, maternal age, maternal prepregnancy BMI, maternal education, maternal gestational smoking, and maternal gestational hypertension/preeclampsia.

^c^ Adjusted according to model 1 (newborn sex, ethnicity, gestational age, birth weight, maternal age, maternal prepregnancy BMI, maternal education, maternal gestational smoking, and maternal gestational hypertension/preeclampsia) with additional adjustment for child age, child weight, child height at follow-up, and household smoke exposure.

While accounting for all aforementioned covariates, newborns with longer TL (both cord blood and placenta) showed lower odds of having a high blood pressure (90th percentile or above of SBP or DBP using the 2017 AAP age-, sex-, and height-specific BP percentiles) in childhood (Table 3). A 1-IQR increase cord blood TL was associated with a lower adjusted OR of 0.72 (95% CI, 0.53 to 0.98; *P* = .03) and a 1-IQR increase placental TL was associated with a lower adjusted OR of 0.69 (95% CI, 0.52 to 0.92; *P* = .01).

**Table 3.** Newborn Telomere Length and Odds for High Blood Pressure in Children Aged 4 to 6 Years

Model	OR (95% CI)^a^	*P* value
Cord blood TL (n = 485)		
Unadjusted model	0.79 (0.59-1.07)	.13
Model 1^b^	0.75 (0.55-1.02)	.07
Model 2^c^	0.72 (0.53-0.98)	.03
Placenta TL (n = 464)		
Unadjusted model	0.71 (0.53-0.94)	.02
Model 1^b^	0.70 (0.52-0.94)	.02
Model 2^c^	0.69 (0.52-0.92)	.01

Abbreviations: OR, odds ratio; TL, telomere length.

^a^ Estimates presented as an OR for high childhood blood pressure as defined using the 2017 American Academy of Pediatrics guidelines for a 1-IQR increase cord blood or placental TL.

^b^ Adjusted for newborn sex, ethnicity, gestational age, birth weight, maternal age, maternal prepregnancy body mass index, maternal education, maternal gestational smoking and maternal gestational hypertension/preeclampsia.

^c^ Adjusted according to model 1 (newborn sex, ethnicity, gestational age, birth weight, maternal age, maternal prepregnancy body mass index, maternal education, maternal gestational smoking and maternal gestational hypertension/preeclampsia) with additional adjustment for child age, child weight, child height at follow-up, and household smoke exposure.

## Discussion

The core finding of this study was that newborn TL was associated with blood pressure indicators early in life. Children born with longer TL (reflected by cord blood and placental TL) displayed a lower diastolic blood pressure and MAP at the age of 4 to 6 years. This shows the potential of TL at birth as a biologically relevant parameter to monitor healthy vs unhealthy aging trajectories.

Telomeres contain thousands of tandem repeated TTAGGG sequences that get lost after each cell division due to incomplete DNA replication. TL represents the replicative history of cells and when telomeres critically shorten or become dysfunctional, cellular senescence is induced.^28^ Experimental studies show that dysfunctional telomeres relate to dilated cardiomyopathy, ventricular wall remodelling,^6^ and endothelial cell senescence in atherosclerosis.^29^ Telomere shortening is observed in human vascular endothelial cells in areas of high hemodynamic stress compared with endothelial cells in the vascular system with low hemodynamic stress.^30^ Vascular smooth muscle cells in atherosclerotic plaques show shorter telomeres than in normal vessels.^31^ These observations suggest that telomere biology and cardiac-specific cell aging in cells factor into blood pressure control. Furthermore, genome-wide association studies of adult leukocyte TL (LTL) identified 7 single-nucleotide variations (*ACYP2*, *NAF1*, *OBFC1*, *RTEL1*, *TERC*, *TERT*, *ZNF208*) linked with LTL that mutually project towards an increased risk for coronary artery disease.^7^

Leukocyte TL is commonly used in epidemiological settings, and may be an overall indicator of the telomeric state of an individual. Because of the high intra-individual synchrony and shortening rate in TL between different tissues in adulthood,^32^ average LTL may provide some information about an individual’s tissue and cell-specific average telomeric state. We show that TL in both cord blood leukocytes and placental cells is associated with blood pressure in early childhood. Whether cord blood TL or placental TL also reflects cardiac cell- and tissue-specific telomere shortening at birth or later life is unknown. Previous research showed that shorter cord telomeres tend to be associated with a higher PDAY score approximately 42 years later in life, but no association with hypertension and blood pressure was observed.^18^ A study including 97 children showed an inverse association between cord blood mononuclear cell TL and SBP in boys after a median follow-up of 4 years, but no association with DBP was observed.^19^ We show associations with DBP and MAP, but associations with SBP were not observed with cord blood TL. Why an association with DBP and not with SBP was observed is still unclear. Differences in SBP and DBP variability may be different in children than in adults. In addition, DBP is a major contributor in secondary hypertension in children, especially in children aged under 6 years.^27,33,34,35^ This indicates the potential important role of DBP very early in life. The importance of elevated childhood blood pressure is further evident from a recent meta-analysis including 39 714 children (age range, 3 to 19 years) that showed that a 1-SD increase in both childhood SBP and DBP results in 71% (OR, 1.71; 95% CI, 1.50-1.95) and 57% (OR, 1.57; 95% CI, 1.37-1.81) higher risk for hypertension in adulthood (age range, 21 to 49 years).^36^ Furthermore, as blood pressure from childhood onwards tracks over time,^1^ it has been shown that elevated childhood blood pressure relates to intermediate markers of cardiovascular disease including increased pulse wave velocity, increased intima-media thickness, and left ventricular hypertrophy.^2,37^ Although the observed associations appear to be small, a population-wide reduction in diastolic pressure by approximately 1 mm Hg is likely to result in a 7% decrease in coronary heart disease and a 10% decrease in stroke later in life,^38^ showing the clinical relevance of a 0.96 to 1.54 mm Hg change in diastolic pressure observed in our study.

As TL tracks from birth to later life^10^ and is associated with blood pressure later in life, our results may give further interpretations to observations that link adult LTL with hypertension^9^ and increased risks for CVD,^4^ in which a part of these observations may be traced back to birth. TL at birth is highly variable,^39^ and understanding the reason for the variation in TL among individuals is crucial for differentiating between causal pathways vs reactive pathways or consequences. As we found out that TL at birth associates with blood pressure at the age of 4 to 6 years, we might think that besides consequential changes, fetal programming may also play a role, but this should be further evaluated. TL, established during gestational life and continuously modified thereafter, might include a signature of inheritance as well as the effects of the intrauterine and extrauterine environments. Therefore TL at birth may be an important biological mediator between genetics, prenatal environmental risk factors, and later life cardiovascular events, and which could provide important insights into the fetal programming of disease hypothesis as postulated by Barker.^40,41^ Besides Barker, Brenner^42^ can be considered as one of the founding fathers of the developmental origin of health and disease hypothesis. He offers a possible explanation for the inverse association between blood pressure and fetal growth by the number of nephrons a person is born with, in which telomeres may have a role in renal growth and function and therefore would associate with later life hypertension.^8,43^ We acknowledge however that large follow-up studies from birth onwards are needed (evaluating TL and cardiovascular changes at different time point throughout life) to further show the potential involvement of early TL and telomere attrition in these observations.

### Strengths and Limitations

Our study has important strengths, including its relatively large sample size. Furthermore, in children and older people alike, blood pressure variability, the white-coat effect, and observer bias limit the reliability of office measurements. Automated techniques of blood pressure measurement may overcome these limitations. We used standardized conditions and multiple automated blood pressure readings that are characterized by high reproducibility and are not subject to digit preference or observer bias. We show consistent findings between blood pressure indicators and TL measured in 2 tissues collected at birth.

Our study also had several limitations. First, although blood pressure tracks over time, individual lifestyle factors and interventions over the life course can change blood pressures later in life^44^ and alter the risks for cardiovascular diseases. Therefore, whether initial TL is also related to hard cardiovascular disease end points and target organ damage later in life is unknown. To address this latter question, long-term follow-up studies are needed. Second, whether TL measured in cord blood and placenta also reflects cardiac tissue- and cell telomere-aging at birth or later in life is unknown. Mean relative cord blood and placental TL as measured in this study was more a systemic indicator of an individual’s telomeric state, and observations made here need to be interpreted within a population-based perspective and not from an individual-based perspective. Furthermore, indications of telomere integrity, telomere dysfunctionalities, telomere damage, or the amount of critically short telomeres that are specific to tissues or cells besides average TL may relate more strongly to specific telomere-related diseases.^28,45,46^ Therefore average TL is more likely an overall health and disease-susceptibility indicator rather than it is a disease-specific indicator. Third, as newborn TL is associated with parental TL,^39^ the contribution of parental TL in the observed associations may warrant further evaluation. Nevertheless, newborn TL contains parental inherited features and nongenetic information that contributes to the observed associations with blood pressure. Finally, our population showed a relatively high prevalence of children with elevated blood pressure according to the 2017 AAP guidelines, which is in line with previous observations.^47^ This is due to the stringent guidelines as a purpose for early detection, potential intervention, and follow-up recommendations when blood pressure is measured on a single day. In this regard, we acknowledge that actual hypertension diagnosis in young children requires repeated blood pressure monitoring over time.

## Conclusions

In this prospective cohort study, newborn TL was associated with blood pressure indicators in children aged 4 to 6 years. TL at birth may be an important early biological marker that links prenatal genetic and nongenetic risk factors with later life health conditions, as in line with the fetal programming of health and disease theory. Additional long-term longitudinal studies with repeated blood pressure measurements over childhood and early adulthood are needed to further examine the role of TL at birth and cardiovascular health trajectories and later life CVD risk. Understanding gene-environmental factors contributing to the initial setting of TL may expand our understanding of the early development of cardiovascular changes.

## Notes

Supplement. **eMethods.** Sample Collection and qPCR Telomere Measurement **eTable 1.** Comparison Characteristics of Analyzed ENVIRONAGE Participants With Not Analyzed Participants and Birth in Flanders Between 1999-2009 **eTable 2.** Blood Pressure Classification According to the 2017 American Academy of Pediatrics Guidelines **eTable 3.** Sensitivity Analysis for the Association Between Newborn Telomere Length and Blood Pressure in Children Aged 4-6 Years
