# Supplementary material for: Association of Newborn Telomere Length With Blood Pressure in Childhood
Source: JAMA Netw Open. 2022 Aug 5;5(8):e2225521. doi: 10.1001/jamanetworkopen.2022.25521 (PMC9356312; doi:10.1001/jamanetworkopen.2022.25521)
Supplement: Supplement. — eMethods. Sample Collection and qPCR Telomere Measurement eTable 1. Comparison Characteristics of Analyzed ENVIRONAGE Participants With Not Analyzed Participants and Birth in Flanders Between 1999-2009 eTable 2. Blood Pressure Classification According to the 2017 American Academy of Pediatrics Guidelines eTable 3. Sensitivity Analysis for the Association Between Newborn Telomere Length and Blood Pressure in Children Aged 4-6 Years [file jamanetwopen-e2225521-s001.pdf]

# Supplemental Online Content

Martens DS, Sleurs H, Dockx Y, Rasking L, Plusquin M, Nawrot TS. Association of newborn telomere length with blood pressure in childhood. *JAMA Netw Open.* 2022;5(8):e2225521. doi:10.1001/jamanetworkopen.2022.25521

**eMethods.** Sample Collection and qPCR Telomere Measurement

**eTable 1.** Comparison Characteristics of Analyzed ENVIRONAGE Participants With Not Analyzed Participants and Birth in Flanders Between 1999-2009

**eTable 2.** Blood Pressure Classification According to the 2017 American Academy of Pediatrics Guidelines

**eTable 3.** Sensitivity Analysis for the Association Between Newborn Telomere Length and Blood Pressure in Children Aged 4-6 Years

This supplemental material has been provided by the authors to give readers additional information about their work.

© 2022 Martens DS et al. *JAMA Network Open.*

**eMethods. Sample collection and qPCR telomere measurement** Umbilical cord blood was drawn immediately after delivery in BD Vacutainer® plastic whole blood tubes with spray-coated K2EDTA (BD, Franklin Lakes, NJ, USA). Blood samples were centrifuged at 3200 rpm for 15 min at room temperature to separate the buffy coat from plasma and erythrocytes. The buffy coat was gently removed using a disposable Pasteur pipette, ensuring minimal plasma and erythrocyte contamination. Buffy coats were stored in cryovials at -80 degrees upon DNA extraction. Complete blood cell counts and differential leukocyte counts are determined using an automated cell counter with flow differential (Cell Dyn 3500, Abbott Diagnostics, Abott Park, IL, USA). Placental biopsies (1 to 2 cm3) were taken directly underneath the chorioamniotic membrane for DNA extraction at the fetal side at approximately 4 cm from the umbilical cord. Care was taken by visual examination and dissection to avoid chorioamniotic membrane contamination. Histological examination of fetal placental biopsies confirmed that the placental sample tissue mainly contained cytothrophoblasts and syncytiotrophoblasts differentiated from trophoblasts. Placental and cord blood leukocyte DNA was extracted using the QIAamp DNA Mini Kit (Qiagen, Inc., Venlo, the Netherlands). DNA quantity and purity was assessed by a Nanodrop 1000 spectrophotometer (Isogen, Life Science, Belgium). DNA was considered pure when the A260/280 was greater than 1.80 and A260/230 greater than 2.0. DNA integrity was assessed by agarose gel-electrophoresis. To ensure a uniform DNA input of 5 ng for each qPCR reaction, samples were diluted and checked using the Quant-iT™ PicoGreen® dsDNA Assay Kit (Life Technologies, Europe). The telomere-specific qPCR reaction mixture contained 1x QuantiTect SYBR Green PCR master mix (Qiagen, Inc., Venlo, the Netherlands), 2 mM dithiothreitol (DTT), 300 nM telg primer (ACACTAAGGTTTGGGTTTGGGTTTGGGTTTGGGTTAGTG T) and 900 nM telc primer (TGTTAGGTATCCCTATCCCTATCCCTATCCCTATCCCTAACA). Cycling conditions used were: 1 cycle at 95°C for 10 min, followed by 2 cycles at 94°C for 15 sec and 49°C for 2 min and 30 cycles at 94°C for 15 sec, 62°C for 20 sec, and 74°C for 1 min and 40 sec. The single-copy gene qPCR mixture contained 1x QuantiTect SYBR Green PCR master mix, 300 nM 36B4u primer (CAGCAAGTGGGAAGGTGTAATCC) and 500 nM 36B4d primer (CCCATTCTATCATCAACGGGTACAA) . Used cycling conditions were: 1 cycle at 95°C for 10 min, followed by 40 cycles at 95°C for 15 sec, and 58°C for 1 min and 20 sec. All measurements were performed in triplicate on a 7900HT Fast Real-Time PCR System (Applied Biosystems) in a 384-well format. After each qPCR a melting curve analysis was performed. On each run, a 6-point serial dilution of pooled buffy coat or pooled placental DNA was run to assess PCR efficiency as well as eight inter-run calibrators to account for inter-run variability. qPCR curves for each sample were visually inspected and when technical problems were detected or triplicates showed too high variability, samples were removed for further analysis (n=18 for cord blood and n=28 for placental TL). Telomeres were measured in two separate batches, and were calculated using qBase (Biogazelle, Zwijnaarde, Belgium). The reliability of our assay was assessed by calculating the interclass coefficient (ICC) with 95% CI of triplicate measures (T/S ratios, T and S measures separately) for both cord blood and placental telomeres using the R package available at the Telomere Research Network website.[1] The inter-assay ICC was 0.94 (95%CI: 0.81 to 0.97) and the intra-assay ICC was 0.95 (95%CI: 0.95 to 0.96).

© 2022 Martens DS et al. *JAMA Network Open.*

**eTable 1. Comparison characteristics of analyzed ENVIR*ON*AGE participants with not analyzed participants and all births in Flanders between 1999-2009**

| Characteristic | | ENVIR*ON*AGE analysed (n=485) | ENVIR*ON*AGE not analysed (n=956) | Flanders (n=606 877)* |
|---|---|---|---|---|
| **Newborn** | | | | |
| Sex | | | | |
| Girls, n | | 52.8% | 46.7% | 48.6% |
| Boys, n | | 47.2% | 53.3% | 51.4% |
| Ethnicity | | | | |
| European, n | | 94.6% | 82.9% | 87.7% |
| Non-European, n | | 5.4% | 17.1% | 12.3% |
| Gestational age, | | 39.0 (37.0-41.0) | 40.0 (38.0-41.0) | NA |
| Gestational age, | | | | |
| <32 | | 0.2% | 0.7% | 0.6% |
| 32-36 | | 5.6% | 4.2% | 5.3% |
| >36 | | 94.2% | 95.1% | 94.1% |
| Birth weight, g | | 3385 (2805-4035) | 3420 (2790-4030) | 3360 (2740-3965) |
| **Maternal** | | | | |
| Age at delivery, yrs | | 30.0 (25.0-36.0) | 29.0 (23.0-35.0) | 29.5 (23.5-35.8) |
| Education | | | | |
| Low, n | | 5.8% | 14.4% | 13.1% |
| Middle, n | | 28.4% | 40.1% | 40.8% |
| High, n | | 65.8% | 45.5% | 46.2% |
| Parity | | | | |
| 1 | | 52.6% | 52.5% | 46.9% |
| 2 | | 37.1% | 34.6% | 34.7% |
| ≥3 | | 10.3% | 12.9% | 18.4% |

Values are presented as median (10-90$^{th}$ percentile) or as frequency (%).
*Data from birth register that comprises all birth from Flanders[2,3] from 1999-2009, n= 606 877 except for gestational age categories (n= 525 635)

© 2022 Martens DS et al. *JAMA Network Open.*

## eTable 2. Blood pressure classification according to the 2017 American Academy of Pediatrics Guidelines.[4]

| BP category | | SBP/DBP percentile[a] |
|---|---|---|
| **Normal BP** | | < 90th percentile |
| **Elevated BP** | | ≥ 90th to <95th percentile or 120/80 mmHg to <95th percentile (whichever is lower) |
| **Stage 1 hypertension** | | ≥ 95th to <95th percentile + 12 mmHg, or 130/80 to 139/89 mmHg (whichever is lower) |
| **Stage 2 hypertension** | | ≥ 95th percentile + 12 mmHg or ≥ 140/90 mmHg (whichever is lower) |

[a]Percentiles are age, sex, and height matched as described by Flynn *et al*., 2017[4], and categories used for ENVIR*ON*AGE are based on the BP categories defined for the age range of 1-13 years in children.

© 2022 Martens DS et al. *JAMA Network Open.*

**eTable 3. Sensitivity analysis for the association between newborn telomere length and blood pressure in children aged 4-6 years.**

| | SBP, mmHg | | | DBP, mmHg | | | MAP, mmHg | |
|---|---|---|---|---|---|---|---|---|
| | difference (95%CI) | p value | | difference (95%CI) | p value | | difference (95%CI) | p value |
| **Cord blood TL models** | | | | | | | | |
| Main model | –0.45 (–1.40, 0.50) | 0.35 | | –1.54 (–2.36, –0.72) | <0.001 | | –1.18 (–1.89, –0.46) | 0.0013 |
| Boys (n=229) | –0.70 (–2.11, 0.70) | 0.32 | | –1.59 (–2.81, –0.38) | 0.010 | | –1.30 (–2.36, –0.23) | 0.017 |
| Girls (n=256) | –0.29 (–1.63, 1.05) | 0.67 | | –1.63 (–2.78, –0.47) | 0.0061 | | –1.18 (–2.20, –0.16) | 0.023 |
| Excl. preterm birth[a] (n=457) | –0.36 (–1.34, 0.62) | 0.47 | | –1.62 (–2.47, –0.78) | <0.001 | | –1.20 (–1.94, –0.46) | 0.0015 |
| Excl. overweight[b] (n=419) | –0.25 (–1.27, 0.78) | 0.64 | | –1.44 (–2.34, –0.53) | 0.0019 | | –1.04 (–1.82, –0.24) | 0.0092 |
| Main + child BMI[c] | –0.47 (–1.43, 0.50) | 0.34 | | –1.56 (–2.39, –0.74) | <0.001 | | –1.20 (–1.92, –0.47) | 0.0012 |
| Main + date and season FU | –0.36 (–1.33, 0.61) | 0.46 | | –1.21 (–2.03, –0.39) | 0.0039 | | –0.93 (–1.65, –0.21) | 0.011 |
| Main + maternal BP (n=459) | –0.35 (–1.31, 0.61) | 0.48 | | –1.54 (–2.36, –0.72) | <0.001 | | –1.14 (–1.86, –0.43) | 0.0017 |
| **Placenta TL models** | | | | | | | | |
| Main model | –0.71 (–1.59, 0.16) | 0.11 | | –0.96 (–1.72, –0.21) | 0.012 | | –0.88 (–1.54, –0.22) | 0.0090 |
| Boys (n=217) | –1.00 (–2.28, 0.28) | 0.12 | | –0.88 (–1.99, 0.22) | 0.12 | | –0.92 (–1.88, 0.04) | 0.059 |
| Girls (n=247) | –0.48 (–1.73, 0.77) | 0.45 | | –1.26 (–2.35, –0.17) | 0.024 | | –1.00 (–1.96, –0.04) | 0.042 |
| Excluding preterm birth[a] (n=440) | –0.65 (–1.56, 0.26) | 0.16 | | –0.92 (–1.70, –0.14) | 0.021 | | –0.83 (–1.51, –0.15) | 0.017 |
| Excl. overweight[b] (n=402) | –0.75 (–1.72, 0.22) | 0.13 | | –0.93 (–1.77, –0.10) | 0.029 | | –0.82 (–1.55, –0.08) | 0.030 |
| Main + child BMI[c] | –0.65 (–1.53, 0.23) | 0.15 | | –0.95 (–1.70, –0.20) | 0.014 | | –0.85 (–1.51, –0.19) | 0.012 |
| Main + date and season FU | –0.60 (–1.49, 0.29) | 0.18 | | –0.73 (–1.49, 0.02) | 0.056 | | –0.69 (–1.35, –0.03) | 0.042 |
| Main + maternal BP (n=439) | –0.68 (–1.57, 0.21) | 0.14 | | –1.12 (–1.87, –0.37) | 0.0035 | | –0.99 (–1.65, –0.34) | 0.0031 |

Estimates presented as a difference (95%CI) in blood pressure indicator (mmHg) for an IQR longer cord blood or placental TL. Main model adjusted for newborn sex, ethnicity, gestational age, birth weight, maternal age, maternal pre-pregnancy BMI, maternal education, maternal smoking during pregnancy, maternal gestational hypertension and preeclampsia, child age, child weight, child height at follow-up and household smoke exposure during follow-up.
[a]Preterm birth is defined as gestational age less than 37 weeks.
[b]Child overweight (including obesity) as defined by age- and sex-specific BMI cut-offs according to the IOTF
[c]In Models adjusting for child BMI we excluded child height and child weight.

© 2022 Martens DS et al. *JAMA Network Open.*

## eReferences

1. Eisenberg DT, Nettle D, Verhulst S. How to calculate the repeatability (ICC) of telomere length measures. 2020. https://trn.tulane.edu/resources/study-design-analysis/ (accessed 30 January 2021).
2. Cox B, Martens E, Nemery B, Vangronsveld J, Nawrot TS. Impact of a stepwise introduction of smoke-free legislation on the rate of preterm births: analysis of routinely collected birth data. *Bmj* 2013; **346**: f441.
3. Winckelmans E, Cox B, Martens E, Fierens F, Nemery B, Nawrot TS. Fetal growth and maternal exposure to particulate air pollution--More marked effects at lower exposure and modification by gestational duration. *Environmental research* 2015; **140**: 611-8.
4. Flynn JT, Kaelber DC, Baker-Smith CM, et al. Clinical Practice Guideline for Screening and Management of High Blood Pressure in Children and Adolescents. *Pediatrics* 2017; **140**(3).
